# Bacterial-fungicidal vine disease detection with proximal aerial images

**DOI:** 10.1016/j.heliyon.2024.e34017

**Published:** 2024-07-08

**Authors:** Delia Elena Székely, Darius Dobra, Alexandra Elena Dobre, Victor Domşa, Bogdan Gabriel Drăghici, Tudor-Alexandru Ileni, Robert Konievic, Szilárd Molnár, Paul Sucala, Elena Zah, Adrian Sergiu Darabant, Attila Sándor, Levente Tamás

**Affiliations:** a Department of Horticultural Sciences, University of Agricultural Sciences and Veterinary Medicine, Cluj-Napoca, Romania; b Department of Parasitology and Parasitic Diseases, University of Agricultural Sciences and Veterinary Medicine, Cluj-Napoca, Romania; c Computer Science, Babes Bolyai University, Cluj-Napoca, Romania; d Department of Parasitology and Zoology, University of Veterinary Medicine, Budapest, Hungary; e HUN-REN-UVMB Climate Change, New Blood-Sucking Parasites and Vector-Borne Pathogens Research Group, Budapest, Hungary; f Automation Department, Technical University of Cluj-Napoca, Cluj-Napoca, Romania

**Keywords:** Vine disease detection, Image processing, UAV, *Plasmopara viticola*, Neural networks

## Abstract

Vine disease detection is considered one of the most crucial components in precision viticulture. It serves as an input for several further modules, including mapping, automatic treatment, and spraying devices. In the last few years, several approaches have been proposed for detecting vine disease based on indoor laboratory conditions or large-scale satellite images integrated with machine learning tools. However, these methods have several limitations, including laboratory-specific conditions or limited visibility into plant-related diseases. To overcome these limitations, this work proposes a low-altitude drone flight approach through which a comprehensive dataset about various vine diseases from a large-scale European dataset is generated. The dataset contains typical diseases such as downy mildew or black rot affecting the large variety of grapes including Muscat of Hamburg, Alphonse Lavallée, Grasă de Cotnari, Rkatsiteli, Napoca, Pinot blanc, Pinot gris, Chambourcin, Fetească regală, Sauvignon blanc, Muscat Ottonel, Merlot, and Seyve-Villard 18402. The dataset contains 10,000 images and more than 100,000 annotated leaves, verified by viticulture specialists. Grape bunches are also annotated for yield estimation. Further, tests were made against state-of-the-art detection methods on this dataset, focusing also on viable solutions on embedded devices, including Android-based phones or Nvidia Jetson boards with GPU. The datasets, as well as the customized embedded models, are available on the project webpage.^2^

## Highlights

• Review of Vine Disease Detection.
• Remote sensing with UAV.
• Machine learning based methods.

## 1 Introduction

Europe has the highest vineyard density globally, while Romania ranks 6th in European wine production, having a long history of winemaking [1]. Disease-induced losses are prevalent in the viticultural sector, requiring a continuous protection approach. This involves applying fungicides/pesticides uniformly and periodically in the vineyards. In some major production regions, the number of treatments per season exceeds twelve. Early detection of grapevine disease symptoms is pivotal for selective treatment targeting, which prevents and controls infection development and its epidemic dissemination to other plots or to the whole vineyard. The success of AI-based tools for viticulture and winemaking depends on the availability and quality of target data or ground truth, as well as the ability of machine learning (ML) models to learn from new data. In recent years, numerous studies have explored the imagery of unmanned aerial vehicles (UAVs) for viticulture [2], [3], [4]. Most of these studies concentrated on single applications at particular phases of the vegetative cycle, such as detecting missing plants, mapping vigour, measuring photosynthesis activity, estimating canopy height, assessing plant water status and identifying diseases [5], [6], [7], [8].

Due to the increasing popularity of computer vision and deep learning methods, precision agriculture has also experienced a paradigm shift in recent decades. Precision viticulture represents a relevant sector within this domain, as reflected by the many research articles focusing on this topic over the last few years. Most of these works focus on creating a dataset on infected plants, a crucial part of any machine-learning application for precision agriculture. A well-constructed dataset includes sufficiently varied data with consistent annotation. Due to the periodic nature of most agricultural goods, including grapevines, the dataset creation phase is limited to only a few months each year. The challenges associated with the considerable efforts in creating these datasets lead to unrepresentative models with a small or very limited variety of datasets [9].

By creating and sharing these datasets, the quality of the papers could be considerably enhanced. Only a few datasets are publicly available, even though most researchers conduct field measurements. Furthermore, with the advance of different machine learning techniques, even with a small amount of local data, it can be achieved to use the foundation models to fine-tune a good specific model [10].

Precision viticulture includes many challenges, including disease detection, vigour, or yield estimation with specific solutions. Hence, a different dataset is required for each task. A dataset for yield estimation is not useful for disease detection, and vice versa. This further underpins the importance of sharing datasets with appropriate annotations. The use case can be developed even further, considering data acquisition devices, platforms, and special circumstances, such as the use of autonomous vehicles, specific camera types, and distances from the plants [11].

This paper aims to create a large unmanned aerial vehicle-based proximal benchmark dataset for detecting vine disease. The main motivation is to provide a benchmark dataset for vine disease detection using images of commercial-grade UAVs, which is still missing from the main literature. Although similar projects exist, the current variant with a 10 K range of images and a 100 K range of annotation boxes is by order of magnitude larger than the existing datasets. This is crucial for validating different recognition algorithms in various stages and conditions of plants. In addition, the great spatial variety for image sampling (including several countries such as Romania, Hungary, Serbia, Slovakia, and France) makes the current dataset generic enough for evaluation purposes compared to single-point datasets such as [12]. With a great variety of vine diseases in focus, the proposed dataset is suitable for multi-purpose evaluation as well compared to a single type of diseases existing in the literature [13], [14]. By considering deploying custom disease detection models on embedded platforms suitable for lightweight UAVs, this work represented a pioneering approach to precision agriculture. The dataset and the tested algorithms are publicly available on the project's website, supporting reproducible research in this domain.

Most of the dataset was acquired from Romania; however, images from 13 different vineyards were also included from 4 other countries. Later, an evaluation was performed using the dataset using two methods based on deep learning, both based on a popular YOLO detection algorithm (YOLO) [15]. One method is detection-based, and one is segmentation-based. We use box labels for detection, and for the segmentation task, we interpret those boxes as rectangle segmentation instances. The dataset and useful preprocessing scripts and pre-trained models are available on the project website.^3^

It is pertinent to acknowledge that the majority of machine learning (ML) methodologies presuppose the utilization of supervised learning paradigms for this particular task (detection and segmentation), thereby necessitating an extensive corpus of annotated data (minimally encompassing instances of both diseased and non-diseased grape vines) to cultivate a precise model. Semi-supervised techniques are employed to mitigate the challenges posed by a lack of labelled data, capitalizing on the presence of unlabeled data to bolster outcomes. Nevertheless, due to the constraints imposed by limited datasets, such approaches encounter complications when confronted with novel patterns within the target dataset for classification that were not present during the model's training phase. In essence, most classification algorithms demand a comprehensive enumeration of all potential problem classes in advance, and their performance deteriorates when a new class materializes within dynamic environments. Addressing this pivotal issue constitutes the central focus of the present study.

The financial aspects of the different field monitoring techniques are also relevant. In Table 1, we summarized a few aspects of these surveys, starting from the *setup cost*, which can be neglected if the user does not own the equipment but rents it. The *Care cost* can be viewed as renting cost, salaries, repair, or energy usage. Further advantages and disadvantages are also mentioned. In this table we listed acquisition vehicles, however most of them could be included in more than one category: Manual image acquisition can be proximal or extreme proximal, as a drone can be considered either proximal or remote sensing. Additionally, each base can be equipped with specific sensors regarding their types and qualities.

**Table 1:** Comparison of different visual monitoring approaches.

Approach	Initial cost	Running cost	Advantages	Disadvantages
Satellite	Low	Very Low	Very large area and autonomy	Very infrequent, very low details, depends on the weather conditions
Fixed winged drones	High	Medium	Large area	Infrequent, low details, hours of autonomy
Rotary drones	Medium	Medium	Frequent, medium details	Medium area, minutes of autonomy
Ground robots	Medium	Low	Frequent, high details	Small area, varying autonomy, depends on the weather conditions
Manual inspection	Low	Low	Very high details	Very small area, infrequent

Several studies have leveraged high-definition imagery acquired via satellites, aircraft, terrestrial machinery, and Unmanned Aerial Vehicles (UAVs) to diagnose crop diseases. The expansive reach of satellites and aircraft enables rapid surveying of extensive tracts of land. Nevertheless, these platforms are often hampered by suboptimal spatial and temporal resolution in their imagery, starkly contrasting to UAVs. They are vulnerable to meteorological conditions that may impede overflight operations. Consequently, applying aerial remote sensing through drones equipped with advanced visual systems presents a cost-effective and efficient methodology for agronomists to monitor and identify plant pathologies across diverse agricultural settings, ranging from small-scale greenhouses to expansive farmlands. While drones are recognized for their high efficiency, cost-effectiveness, adaptability, precision, and rapid deployment at the field scale, their restricted flight endurance renders them impractical for data collection over expansive areas. Therefore, the meticulous selection of an appropriate drone model and the corresponding sensors, software, algorithms, and configuration parameters is imperative to optimize performance outcomes in aerial data acquisition tasks.

### 1.1 Available datasets in the literature

The methods of precision viticulture can be divided into two major categories: proximal sensing and remote sensing [16]. Proximal sensing methods focus on visual analysis of individual organs, while remote sensing focuses on the overall status of the plant. However, there is no strict distance limit where the two categories alter: methods based on organ-level grapevine assessment are considered for proximal sensing because this task necessitates relatively close-range image acquisition. On the other hand, vineyard-level status analysis is bound to higher altitude image capturing. This differentiation is also reflected in the equipment used. Proximal sensing methods prefer handheld or tractor-mounted RGB cameras [17], while infrared and other spectral cameras are quite rare in this context [18]. In remote sensing, on the other hand, multispectral and hyperspectral cameras mounted on various aerial vehicles, such as planes, drones, or satellites, are preferred against conventional RGB cameras [19], [20].

Only a limited number of methods explore this idea. Torres-Sánchez et al. [21] fly at an altitude of 10 - 15 meters to reconstruct the plant structures, while Su et al. [22] calculate the canopy density. Music et al. [23] fly their drones at heights of 20 - 25 meters to detect fluorescence dorée using *Faster-RCNN* [24]. Although this example can be considered borderline remote sensing because, due to the distance, the plants are analyzed in their entirety, and not by individual organs, the altitude is still lower than usual, such as [25], whose method requires to use multispectral cameras, because a conventional RGB camera would not be suitable. On the contrary, del-Campo-Sanchez et al. [26] fly over an altitude of 80 meters to detect the impact of Jacobiasca lybica, however, only with the help of point clouds and orthoimages, which is similar to the approach of Wang et al. [27], or to Zottele et al. [28], who add multispectral data to the equation. Kerkech et al. [29] go even higher (about 110 meters); however, they accompany their RGB sensor data with infrared information to create a mildew detector. In a subsequent article, Kerkech et al. [30] experiment with a lower altitude of 20 - 25 meters, adding near-infrared images and depth maps. Ouhami et al. [31] work at the same altitude while combining meteorological data with RGB, near-infrared, and depth images. Each vineyard has a specific environment with different species; hence, most researchers start their work by creating a small dataset in their local vineyard. However, this procedure takes time and might not be available to everyone, especially because the grape is a seasonal plant, and expensive autonomous capturing systems are not yet widespread. Some of these small datasets are publicly available, and in Table 2, the vine disease-specific datasets, where the access link is clickable in *pdf*.

**Table 2:** Publicly available datasets for grapevine analysis at the end of 2023.

Name	Description	Link
PlantVillage (2015) Hughes et al. [32] Mohanty et al. [33]	50000 *RGB* images (4000 grape images) for disease detection and classification, multiple plant species are included (pepper, tomato, grape, etc) in a *laboratory*, grapes with categories: *healthy*, *black rot*, *esca*, and *leaf blight*	Link
Škrabánek and Runarsson (2015) [34]	5 *RGB* images of *Welschriesling* canopy photographed from a tractor, with a perpendicular view	Link
GrapeCS-ML (2018) Seng et al. [2]	2300 *RGB* images for segmentation and yield estimation	Link
Salento Grapevine Yellows dataset (2018) Cruz et al. [35] Ampatzidis et al. [36]	4700 *RGB* images for disease detection and classification (*powdery mildew*, *grapevine yellow*, *stictocephala bisonia*, *leafroll*, *healthy*, *black rot*, *esca*, and *leaf blight*) on laboratory environment	Link
del-Campo-Sanchez et al. (2019) [26]	1 orthoimage for *Jacobiasca lybica* detection from an altitude of more than 80 m	Link
CR2 Dataset (2020) Coviello et al. [37]	17 *RGB* proximal images for yield estimation with berry annotations	Link
Embrapa WGISD (2020) Santos et al. [38]	300 *RGB* images for berry detection using masks and bounding boxes	Link
Abdelghafour et al. (2021) [13]	99 *RGB* flashlight illuminated images for disease detection (*downy mildew*) and segmentation	Link
Aghi et al. (2021) [39]	500 *RGB* images for canopy segmentation and navigation with binary canopy masks	Link
ESCA-dataset (2021) Alessandrini et al. [40]	1700 proximal *RGB* images for *esca* detection	Link
AI4Agriculture Grape Dataset (2021) Morros [41]	250 *RGB* images for grape detection and yield estimation, including Aruco codes	Link
Grapevine Leaves (2021) Vlah [42]	1000 proximal *RGB* images for classification	Link
Zabawa et al. (2021) [43], [44]	42 flashlight illuminated *RGB* images for berry detection using binary mask and artificial background	Link
Buds-Dataset (2022) Apostolidis et al. [45]	100 *RGB* images for segmentation and pruning during the dormant period	Link
GrapevineLeaves ImageDataset (2022) Koklu et al. [46]	500 *RGB* images for vine species classification on individual, flat-pressed leaves	Link
GreenAI (2022) Barros et al. [47]	3 multispectral and 3 orthoimage + *DSM* for remote sensing	Link
S3Cav VineyardDataset (2022) Casado-García et al. [48]	400 *RGB* images for segmentation of bunches, poles and leaves described by masks	Link
wGrapeUNIPD-DL (2022) Sozzi et al. [49]	373 proximal *RGB* images for yield estimation	Link
VitiVisor Vineyard Datasets (2022) Collins et al. [50]	many *RGB* images for segmentation covering the life cycle of multiple plants, including annotations for a few images	Link
Ariza-Sentís et al. (2023) [51]	40 *RGB* videos for object detection and object tracking using an *UAV*	Link
GrapeNet (2023) Barbole et al. [52]	11000 *RGBD* images for yield estimation in real and laboratory environment, including depth information	Link
3D2Cut (2023) Gentilhomme et al. [53]	1511 *RGB* images for structure estimation with artificial background	Link
Grapevine Bunch Condition Detection Dataset (2023) Pinheiro et al. [54]	968 *RGB* images for bunch detection (healthy, damaged)	Link
Grapevine Bunch Detection Dataset (2023) Pinheiro et al. [54]	968 *RGB* images for bunch detection	Link
Tardif et al. (2023) [12]	1483 flashlight illuminated *RGB* images for *esca* and *flavescence dorée* detection with binary masks for shoot and bunch segmentation	Link
Vélez et al. (2023) [55]	16500 multispectral images for *remote sensing* and *botrytis* detection	Link
Roboflow Universe	Many labelled images for different domains, including grapevine diseases too	Link

Although these datasets are promising for specific vine species, regions, or leaf maturity, none represents a generic and comprehensive variant for a close-range, multi-regional, and seasonal dataset. In the current manuscript, we propose a close-range, high-resolution UAV image-based dataset for vine disease detection that covers the gap between the real needs of precision agriculture and existing public datasets. The manuscript is structured as follows: the *Introduction* section is followed by the *Materials and methods* part, which covers the study areas and the collection method details presented in this work. Further image labelling and model architecture-related parts are discussed. In the *Results* section, the focus is on VDD and the deployment on embedded devices, followed by a discussion related to the current approach's ability. Finally, the paper is concluded with the main results and future directives in the *Conclusion*.

## 2 Materials and methods

### 2.1 Proposed methodology

One of the main benefits of remote sensing is that aerial vehicles can cover a larger area in a given time without being hindered by uneven ground surfaces [56]. Unmanned aerial vehicles (UAVs), such as drones, are examples of this scenario when they fly at a high altitude, capturing orthographic images. This saves time and costs both for researchers and vineyard owners as well. However, this kind of remote image is usually bounded by camera type and vegetation index calculations [57]. For early-stage disease detection, however, proximal detection is optimal. A drone flying a few meters above the canopy can provide high-quality RGB images for proximal sensing, which is essential for early-stage disease detection.

To sum up, most UAV-based methods are restrained to convert the RGB data into structural data or to integrate additional sensors. Conventional RGB-based image processing is rarely used for disease detection. Furthermore, yield estimation and berry detection are quasi-nonexistent, probably because grape bunches are situated on the bottom of the canopy and are occluded from a high altitude. Therefore, this UAV-based dataset is nominated to bridge the gap between ground vehicle-based applications and remote sensing applications for vine disease detection.

This work proposes creating a multi-area, multi-season, multi-species, and multi-disease vineyard dataset using close proximity images from low-altitude flight drones (1-3 meters above the vineyard). The main purpose is to create a benchmark dataset with labelled instances for disease detection using modern machine learning-based approaches.

The methodology considered for this dataset acquisition included the following aspects: at least five types of grapes are included to ensure a representative sample; five different countries in the moderate climate region are included to ensure geographical and climate data variance; and the period is from May to October to cover the most common disease installation periods.

The adopted methodology for image labelling and annotation is similar to the common machine learning ones: high-resolution images with a windowing or downsampling approach using semi-automated labelling tools for annotation and post-processing and manual labelling with a team of 9-15 people. The following subchapters discuss all the details regarding the different aspects of the methodology.

### 2.2 Study area

**Figure 1:**
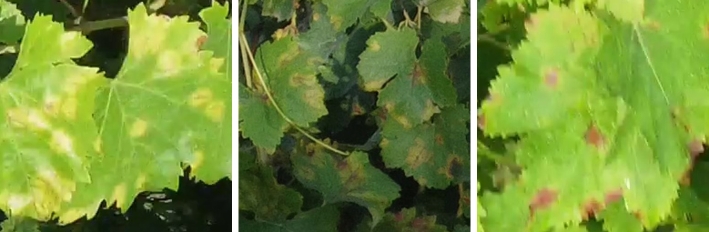
Examples of Downy mildew affected leaves in the dataset.

**Figure 2:**
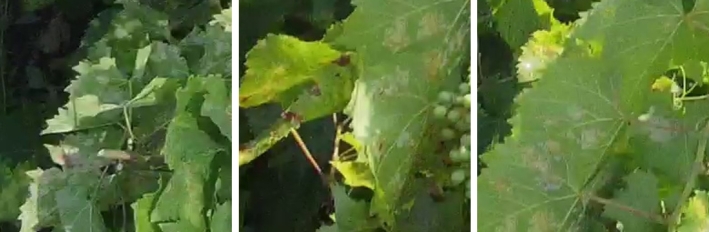
Examples of Powdery mildew affected leaves in the dataset.

**Figure 3:**
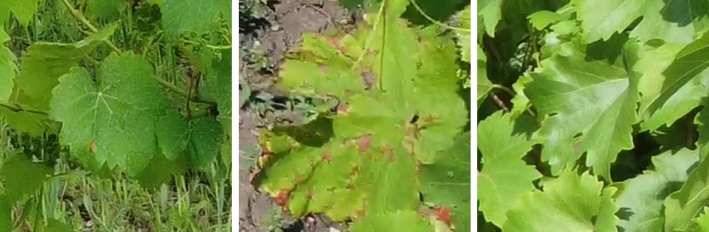
Examples of Black rot affected leaves in the dataset.

**Figure 4:**
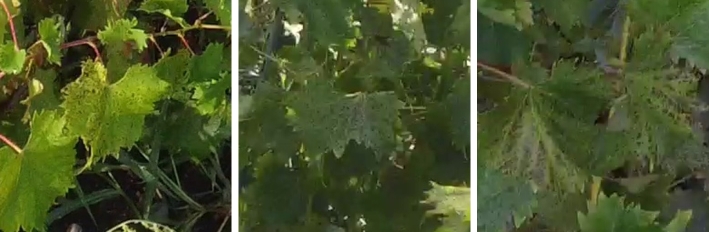
Examples of Excoriosis and anthracnose affected leaves in the dataset.

Grapevine diseases affect the health and longevity of the plants and, consequently, the quality of the wines. An intelligent monitoring system for automatic inspection can assist farmers, enabling rapid detection of diseases at different development stages [58]. In our work, the focus was mainly on the local collection of grapevines of the University of Agricultural Sciences and Veterinary Medicine of Cluj-Napoca, which has a collection of 68 grape varieties, including noble vines, interspecific hybrids, table grapes, and rootstocks, located on a hill. The collection covers an area of 1.5 hectares, with a planting density of 5000 plants per hectare. Multiple drone surveys were conducted during the vegetative period of 2023 (May - October), under different weather conditions (sunny, cloudy) and times of the day (morning, noon, afternoon). The high precipitation and large temperature fluctuations in July and August favoured the establishment and rapid spread of various grapevine diseases such as downy mildew (*Plasmopara viticola* - Fig. 1), powdery mildew (*Erysiphe necator* - Fig. 2), black rot (*Guignardia bidwellii* - Fig. 3), anthracnose (*Elsinoë ampelina*), and excoriosis (*Phomopsis viticola*(Sacc.) - Fig. 4).

In addition to these diseases, mites (*Tetranychus urticae* Koch) and phylloxera (*Daktulosphaira vitifoliae*) caused foliar damage, which had a negligible impact. These diseases were captured in images and videos at different stages of development. To achieve a more homogeneous and objective characterization of the grapevine diseases, data collection was performed by aerial surveys in various locations within and outside Romania. One of these locations was the “Vinea Apoldia Maior Research Center” of the University of Agricultural Sciences and Veterinary Medicine from Cluj-Napoca, located in Apoldu de Sus in Sibiu County. This vineyard is a 65-hectare collection of noble wine grape varieties belonging to the Sebes-Apold Controlled Designation of Origin region. The infection rate in this area was lower due to favourable pedoclimatic conditions.

**Figure 5:**
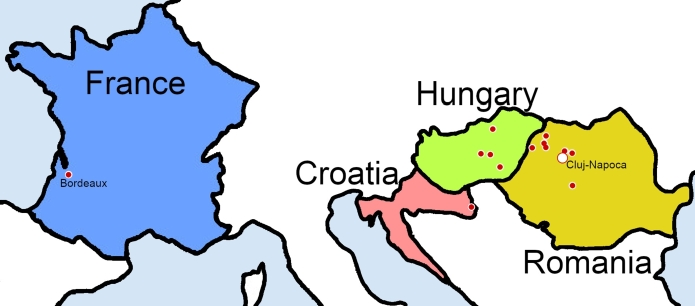
The locations of the dataset acquisition campaigns.

Surveys carried out in Romania cover vineyards in the wine regions of the Transylvanian Plateau (Lechinţa, Sebeş-Apold, Bonţida), Crişana and Maramureş (Şimleu Silvaniei, Carastelec, Diosig, Beltiug). The international surveys were conducted in Hungary in the regions of Kecskemét (at the vineyard of the Hungarian University of Agriculture and Life Sciences Institute for Viticulture and Oenology - MATE), Szűcsi (in the Matra mountain range), Lakitelek, Székkutas. Flight campaigns were also conducted in Croatia at Ilok, and in France at Bordeaux (at the Château Luchey-Halde winery). Fig. 5 presents the geographical placement of the locations, while Table 3 provides specific information about the period and location of the different sub-datasets.

**Table 3:** Information about the vine disease dataset. The name describes the dataset's location, along with the flight campaign periods and coordinates of an image from the respective subset.

Name	Period	Coordinates
Apoldu	July 31	N 45.8378^∘^ - E 23.8531^∘^
Beltiug	May 06	N 47.5379^∘^ - E 22.8579^∘^
Bontida	June 09	N 46.8746^∘^ - E 23.9317^∘^
Bordeaux	September 26	N 44.8199^∘^ - W 0.6309^∘^
Carastelec	September 17	N 47.3065^∘^ - E 22.6868^∘^
Cluj	June 01 - October 03	N 46.7593^∘^ - E 23.5714^∘^
Diosig	September 17	N 47.2889^∘^ - E 22.0105^∘^
Ilok	September 16	N 45.1995^∘^ - E 19.3553^∘^
Kecskemet	August 23	N 46.9704^∘^ - E 19.7253^∘^
Lakitelek	August 26	N 46.8825^∘^ - E 19.9942^∘^
Lechinta	October 03	N 47.0591^∘^ - E 24.2459^∘^
Simleu	June 23	N 47.2372^∘^ - E 22.8133^∘^
Szekkutas	September 17	N 46.4854^∘^ - E 20.5018^∘^
Szucsi	August 24	N 47.8068^∘^ - E 19.7653^∘^

Downy mildew infections affect all*Vitis vinifera* cultivars and many interspecific *Vitis* hybrids and are one of the most destructive diseases of grapevines, especially in regions with warm and humid growing seasons [59], representing an important limiting factor for grapevine cultivation [60], [61]. Disease detection faces two major challenges: one is to classify a plant with multiple diseases, and the other is to distinguish and classify diseases with similar symptoms, especially at the initial stages of development [14]. Vine phenology and production are influenced by temperature, heat, humidity, and light. The symptoms of the disease can manifest in different parts of the plant, but leaf diagnosis is commonly used. Grapevine diseases cause significant losses, and an early and accurate diagnosis is essential for their management [62]. Downy mildew was the dominant disease in the vineyards of the University of Agricultural Sciences and Veterinary Medicine of Cluj Napoca. The vines had delayed growth in May, and the disease became noticeable when the shoots reached 20-50 cm. The pathogen overwinters mainly as oospores in dead fallen leaves [63], although it can also survive as mycelium in buds, causing the first early infections on the break of the buds and leading to the subsequent development of systemic infection. Downy mildew was the most prevalent disease in July and August due to the high rainfall, favouring its spread and development.

*Plasmopara viticola* affected vines in hot and humid environments and caused oily brown lesions on young shoots, tendrils, and grapes. These lesions spread to all plant organs, leading to browning and necrosis. On mature leaves, infections appeared as medium, angular, yellow spots. Multiple infections resulted in coalescing oily spots on a single leaf. They formed a mosaic pattern and turned brown or reddish-brown [64]. After warm and humid nights, the oily spots produced white sporulation [65]. Severe infections cause partial or complete defoliation.

### 2.3 Collection of proximity aerial data protocol

Data acquisition began in May when the leaves were in the infant stage, only a few centimetres in width, and concluded in October as the leaves began to brown. The primary data acquisition site was in Cluj-Napoca. The rest of the data was collected at various other locations detailed in Section 2.2. Each additional location was visited once at different times throughout the acquisition period.

**Figure 6:**
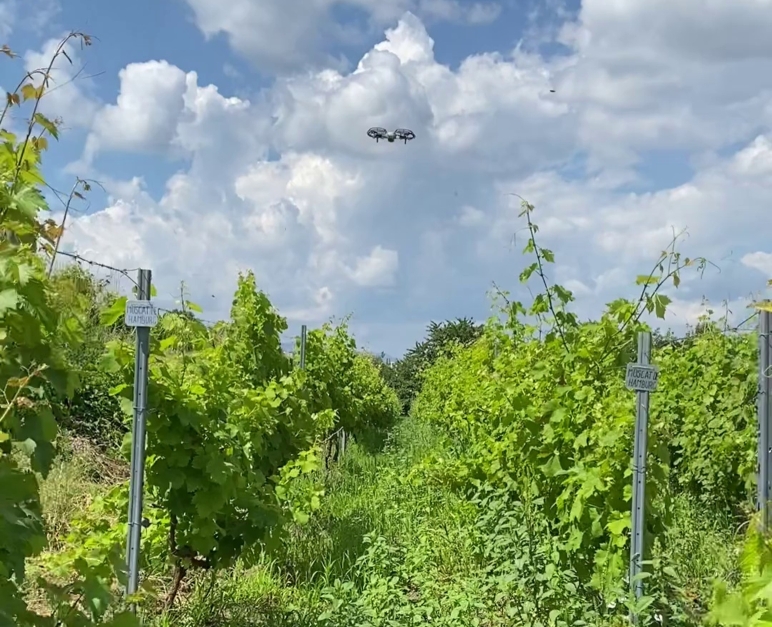
UAV flying in the vineyard and capturing geotagged images for VDD.

Two DJI Mini 2 drones were used for data acquisition, each equipped with a CMOS sensor, an aperture of 2.8, and an $85^{\circ }$ field of view (FOV). The usual setup was the following: The camera gimbal was set at $45^{\circ }$, while the flight altitude was approximately 1-2 meters above the top of the canopies, with a direction parallel to the rows. Exceptions to this methodology exist when the drone flies between rows or is oriented perpendicular to the plant canopy. The camera mode was either Video capture or still photo capture every 2 seconds. From the videos (captured at 3840×2160 pixels @ 24fps, while the still images are captured at 4000×2250 pixels, later resized to 3840×2160 pixels) samples were taken every 50^th^ frame. Most of the images contain the coordinates as metadata, including geolocation data. Certain images were removed according to specific guidelines.: 1) blurry (due to the varying speed or the lighting conditions, the images might appear too blurry to be processable); 2) high altitude (the altitude of the drone might be too high. Therefore the individual leaves are not visible enough); 3) repetition (if the flight speed converges to zero in a given moment, two consecutive sampled images might be too similar, without any significant movement noticeable, hence duplicated data); 4) sensitive data (in a few images might appear a person or a car's license plate). A representative example containing a challenging environment with high grass, direct sunlight, and shadows is presented in Fig. 6. As can be seen, the drone is flying at altitudes less than 10*m* in order to have a sufficiently high resolution of images for VDD.

### 2.4 Image labeling methodology

#### 2.4.1 LabelImg

*LabelImg* is an open-source graphical image labeling tool [66], which generates bounding box labels in either *YOLO*, *PascalVOC*, or *CreateML* format. This tool works on locally stored images, making it the most stable version once set up and started. However, the user must choose the working folder each time the tool is started. Furthermore, due to the large number of images and the multiple annotators, this tool is only viable if a central data server is used to store the images; this setup part can be difficult for generic usage. This annotation tool is recommended for 1-2 person teams who can store images locally without managing rights, batches, and jobs for annotation. For larger teams and datasets, it is suggested that one of the two annotation tools mentioned be considered.

#### 2.4.2 Computer vision annotation tool

*Computer Vision Annotation Tool* (CVAT) [67] is a free and open-source annotation tool originally developed by Intel for computer vision applications. CVAT is used to annotate images and videos and can be accessed either as Software as a Service^4^ or can be self-hosted using the *cvat/ui* or *cvat/server* docker images. In both cases, the Enterprise solution can be purchased, which provides additional features such as integration with Roboflow and advanced analytics. CVAT users need to create an account to use the platform. Users can create organizations that, in turn, can create projects, tasks, and jobs. Each member of the organization can be assigned a role, such as worker, supervisor, maintainer, or owner, and then can be assigned one or more tasks. Tasks can also be shared between users. The annotation tool provides basic features such as image processing using OpenCV, and adding tags, zooming, rotating, and labeling with bounding boxes, polygons, polylines, points, ellipses, and cuboids. In addition, users can create tracks from sequential images or videos. Using tracks, the same object can be annotated through many consecutive images, and frame interpolation can automatically annotate all objects between two annotated frames. CVAT also integrates artificial intelligence tools and models to help with object segmentation and detection tasks. Besides the annotations, custom attributes can be added to each object, such as size (small, medium, big), etc. The AI features were unavailable for the docker server image when writing this article. Additionally, CVAT is not a dataset management tool, meaning that any post-processing of the data (such as image augmentation, cropping, and resizing) needs to be done with another tool, such as Datumaro.^5^ Overall, CVAT is a great tool for annotating sequential images and video frames so that organizations of any size can be managed easily. The fact that the AI tools are only available for the cvat.ai or the user interface docker image limits the tool's usage in our case. For a better job, batch, and right management, the Roboflow tool was opted for.

#### 2.4.3 Roboflow

*Roboflow*^6^ is a shareware platform designed to help users design, build and re-evaluate datasets through the platform workspace. Anyone can use this by uploading a batch of images or a video via *Web* or *API*. The image data can be filtered, segmented, preprocessed, and can be accompanied by tags or augmentation with metadata, location of the image, or split between train/test/validation. The most valuable feature of this web application is the ease of labelling. Through the ability to collaborate on the platform, any user can split the work and annotate their part of the work. As for annotation, it can be done with bounding box labels or by bonding any object with smart polygons. The feature can also be enhanced by the assist label, which can be used after the user has a generated model, or if he wishes to select simple objects like cars, phones, or even people, it can be done with *SAM* (Segment Anything by Meta). Another key feature is the ability to develop, improve, or manage the models created in the platform with the dataset or model added. After the models are trained in a rough mode, more accurate training can be performed, which is helpful with all the metrics offered afterwards. Finally, by using *Roboflow* an option is available to deploy the model through an API endpoint, use it directly in the browser, or deploy directly on IOS, Nvidia Jetson, Personal Web Platform, or Cloud. The unique features of the platform, coupled with the ability to collaborate and divide work among a multidisciplinary team with different operating systems, distinguished *Roboflow* from other programs. This compatibility and teamwork facilitation were the primary reasons for its selection.

On *Roboflow*, the previously acquired and filtered images were divided into smaller batches and assigned to individuals. Each image was scrutinized for bacterial or fungicidal symptoms on the leaves, classifying these as *unhealthy* using bounding box-based annotation. Rectangles were drawn around symptomatic leaves, including partially visible ones, to ensure coverage of diseased spots. The platform's capacity to handle various drone movements and lighting conditions, along with its ability to accommodate overlapping labels, facilitated this process. A second class, *grape*, was used to annotate significant grape bunches, with image class counts detailed in Table 4.

**Table 4:** Information about the number of images that contain annotation for infected leaves or not (thus being healthy), the number of images containing grape annotations, and the number of images without any annotation (Null). Since these categories might overlap, the total number of images in the subset is also mentioned.

Name	Healthy	Infected	Grapes	Null	Total
Apoldu	10	13	17	5	23
Beltiug	-	-	-	52	52
Bontida	40	8	15	28	48
Bordeaux	61	323	9	60	384
Carastelec	9	8	2	8	17
Cluj	2076	6879	2419	1289	8955
Diosig	2	47	44	-	49
Ilok	12	33	37	3	45
Kecskemet	9	41	50	-	50
Lakitelek	8	1	1	7	9
Lechinta	22	87	30	17	109
Simleul	48	4	1	47	52
Szekkutas	11	28	28	4	39
Szucsi	12	130	139	-	142
Sum	2320	7602	2792	1520	9974

**Figure 7:**
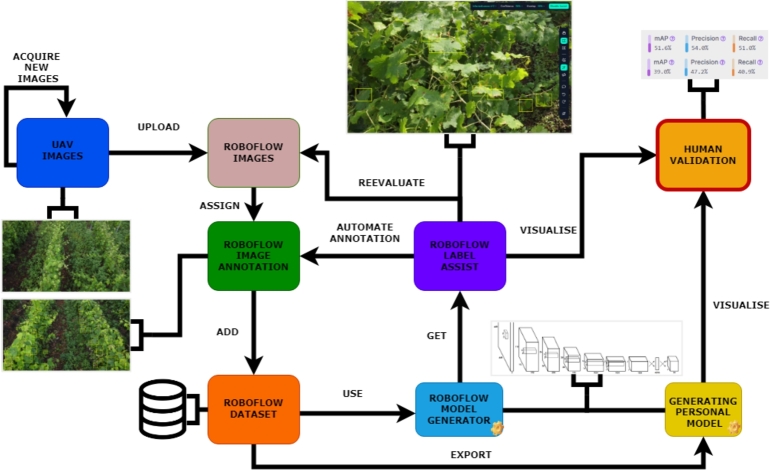
The proposed methodology for the data acquisition, preprocessing, labelling and model completion.

In the Fig. 7 is presented the *Roboflow* Pipeline. As can be seen, the interaction starts with the UAV images, which is a continuous process due to noise, the movement of the drone, and various lighting conditions. Then, the data images gathered previously are filtered and uploaded to the *Roboflow*, where the photos are divided into smaller batches and assigned to a person. After the introductory part is done, the process is moved to the annotation, where on every image, the person is looking for bacterial or fungicidal symptoms on the leaves and tagging this with the class *unhealthy* by drawing a rectangle around each symptomatic leaf. If the leaf is occluded by other leaves, trunks, or branches, it is necessary to mark only the visible part of the leaf. Even if not all the leaves were obvious, the insurance is that a bounding box covered each potentially diseased spot. If the visibility conditions for the leaves were degraded, the person in charge had not marked them. Additionally, a second class was used, *grape*, to tag prominent grape bunches, regardless of their health or stage. In the next step, the annotated data are moved in the dataset, and from this point in the Roboflow platform, if there were enough images, a new model could be generated to help us with the label assist method to automate the annotation part, or to decide if the specific image is good enough for our dataset. Besides utilizing the model on the platform, data can be exported to generate a second model using any desired technique on personal computers, with YoloV8 [15] often chosen similarly to Roboflow. The outcomes from both methods are then compared, and the team's diverse perspectives help determine whether the metrics are satisfactory.

The dataset can be found at,^7^ among pre-trained models and preprocessing scripts. The structure of the dataset is the following: 4 main folders (*images*, *labels*, *labels_grape*, *labels_unhealthy*). Inside the *images* folder, the images are placed following a naming convention: <Location>_<Date>_DJI_<MediaCode: XXXX>_f_<framenumber>.jpg, where the framenumber is optional for images sampled from videos. The *labels* folder contains the labels for all the images, with matching names, except for the file extension, which is ‘.txt’. Here, even the null labels are listed. However, in folders *labels_grape* and *labels_unhealthy*, only one label is listed, respective of the folder name.

### 2.5 Model architecture and training details

Experiments were conducted with the dataset using two different methods to assess the quality of the labelled images. For this purpose, a few Python scripts are provided (at the dataset link) to aid in the data preprocessing. These include resizing the images, splitting the images using a sliding window technique, deleting a specific class from the labels, organizing the images according to the classes, and creating binary masks from labels. On-demand, raw video files, along with a video splitter script, are also available.

The first method for dataset evaluation is the small *YOLOv8* [15]. This architecture is a real-time object detection system that divides the input image into a grid. Each grid cell predicts bounding boxes and class probabilities, enabling simultaneous object localization and classification. Through deeper and more efficient convolutional layers, YOLOv8 improves on this with enhanced speed and accuracy. Its primary advantage is its ability to perform detection quickly and efficiently, making it ideal for applications like detecting unhealthy leaves in various image sizes. In its smaller variants, it is also suited for inference at the edge, making it virtually possible to have disease areas marked and notified in real time.

Different scenarios are considered, including full-size images and smaller sizes using either resize or sliding windows. The second method is a segmentation algorithm based on the *YOLOv8* architecture. For the segmentation task, the label masks are actually the boxes from the detection task.

Converting the labels into binary masks allowed this specific method to be used as an evaluation technique. Both methods underwent evaluation in a single-class mode, facilitating a focused assessment of their effectiveness.

For the experiments, the following metrics are reported: recall, precision, *mean average precision* MAP, Here, the *mean average precision* (MAP) metrics are used, Equation 3. The results for the detection method are presented in Table 6, while for the segmentation method, results are illustrated in Table 5.

(1) $$P=\frac{TP}{TP+FP}$$

(2) $$R=\frac{TP}{TP+FN}$$

(3) $$mAP=\frac{1}{N}\sum_{i=1}^{N}AP_{i}$$

(4) $$IoU=\frac{X\cap Y}{X\cup Y}$$

where the following notations are used: *TP* - true positives, *FN* - false negatives, *FP* - false positives, $P_{i}$ - the maximum precision in the interpolation interval, *N* - number of classes, *R* - recall, *P* - precision, *M* - the data points (pixels), *x* - ground truth value of the pixel, *y* - prediction value of the pixel, *X* - set of the ground truth pixels, *Y* - set of the estimated pixels, $AP_{i}$ is the area under the precision-recall curve (Equation (1) and Equation (2) on class *i*). The difference between mAP50 and mAP50−95 is a different IoU threshold. For the latter, the threshold varies from 0.50 to 0.95.

**Table 5:** Results on the segmentation approach of the unhealthy leaves task using the TOLOv8 nano network, trained and evaluated on *split1* and *split2*. There are also used various image sizes and sliding windows approaches: ds640 - 4k downsampled to 640x360; swds640 - sliding window 4K to 1920x1080 then downsampled to 640x360, swds1280 - sliding window 4K to 1920x1080 then downsampled to 1280x720.

	*split1*			*split2*		
	ds640	swds640	swds1280	ds640	swds640	swds1280
train images	7432	29728	29728	7979	31916	31916
test images	2542	10168	10168	1995	7980	7980
mAP50	0.352	0.494	0.54	0.371	0.483	0.558
mAP50-95	0.162	0.251	284	0.173	0.207	0.289

For this experiment (*split1*), the train and test images are split by position, meaning each image is split according to its location in the video sequence. Since the videos start where the rows begin and end where the rows end, apart from a few exceptions, the frame number in the file's name also refers to the relative location of the image inside the row. Therefore, the first part of each row is included as a training image (frames between 0−3999), while the rest (frames upwards of 4000) are included in the test dataset. In the case when individual images are captured, they are part of the training dataset.

For another experiment, a different split (named *split 2*) was used, where every fifth image is included in the test dataset, while the rest is in the training dataset. The results using this division are presented in Table 8, and Table 5 second column. A third split (named *split 3*) is also presented when the training dataset contains the images from the main location (Cluj-Napoca), while the rest is considered as test data; these results are presented in Table 9, and Table 5 third column. The individual results of this experiment are presented in Table 7. The images were trained at full scale, without the use of sliding windows.

## 3 Results

### 3.1 Vine disease detection results

This Section presents the results for a specific example focusing on the following agricultural image analysis tasks: unhealthy leaves and grape detection. Within this experiment, the climate was considered Mediterranean, with the primary location in Romania. Most species considered for these experiments were Pinot blanc, Pinot Gris, Chambourcin, Fetească regală, Sauvignon blanc, Muscat Ottonel and Merlot. The image acquisition was performed in the summer period, from June to September, with mature leaves with different disease grades. The acquisition was made using a mid-range DJI small-scale (under 250 g) drone with an onboard HD camera and an external Andorid-based processing mobile device. To have a sufficiently large variety of data, different weather and light conditions were considered: from early morning to late afternoon, periods of sun and cloudy to rainy periods were also considered. The average acquisition time was half-hour per day, i.e., the drone's battery lifetime.

The raw data was captured in both continuous video streams and still image formats. The latter proved to be more stable against disturbances. On average, the daily acquired data was around 1 GB, which was later preprocessed and stored on our server. The selected and enhanced images were later considered for training. This image set is also available on the project webpage.

For all the experiments, we use the *YOLOv8* [15] method, capable of processing different image sizes. The *nano* pre-trained model for *YOLOv8* was trained for 100 epochs with a batch size of 4 (for large-size images) and 32 (for small sizes as 640.320). This is the smallest YOLOv8 model, having 3.2M parameters. We model this task as both a detection and a segmentation problem. Firstly, the model predicts the bounding box (rectangle) of the unhealthy leaves or grapes for the detection approach. Secondly, we use the boxes to create segmentation masks for the segmentation approach. Further, we also train and evaluate the models using various image sizes and sliding window dimensions. As presented in the following tables and discussion, the two ways of modelling the task (detection vs segmentation) yield similar results. The major difference in the results is due to the applied image processing methods. In terms of used image size, we use the following notations: 4k - original size, ds640 - 4k downsampled to 640x360; swds640 - sliding window 4K to 1920x1080 then downsampled to 640x360, swds1280 - sliding window 4K to 1920x1080 then downsampled to 1280x720.

For comparison reasons, we run the experiments for *split1* and *split2* for the segmentation approach. Comparing the segmentation (Table 5) and the detection tasks (Table 6, Table 8) for the unhealthy leaves identification we get similar results. For example, for the segmentation task using data *split1* and swds640, the mAP50 is 0.49, which is quite close to the result of the detection experiment with mAP of 0.477. For the *split2*, the segmentation model performs worst with a difference on mAP of 0.034. Having these close results, to enrich the experimental setup, we train the detection model using 4k samples and the segmentation model using sliding windows resized to 1280x720 (swds1280). The best results for this large input size (4k vs. swds1280) are for both the segmentation and detection approaches. In the detection approach where the entire 4k resolution was used, there is an increase of 0.025 mAP50 for *split1* and of 0.026 mAP50 for *split2*. This small difference in performance is also due to the downscale of the sliding windows from a width of 1920 to 1280.

**Table 6:** YOLOv8-split1 (detection): 4K - 3840x2160; ds640 - 4k downsampled to 640x360; swds640 - sliding window 4K to 1920x1080 then downsampled to 640x360. For swds640 preprocessing we also experiment with larger models such as YOLO8-(S)mall, YOLO8-(M)medium and YOLO8-L(arge).

	YOLO8-nano			YOLO8-S	YOLO8-M	YOLO8-L
	4k	ds640	swds640	swds640	swds640	swds640
train images	7432	7432	29728	29728	29728	29728
test images	2542	2542	10168	10168	10168	10168
grape (MAP50)	0.379	0.089	0.119	0.257	0.267	0.277
grape (MAP50-95)	0.164	0.028	0.046	0.093	0.099	0.102
unhealthy (MAP50)	0.565	0.389	0.477	0.523	0.533	0.540
unhealthy (MAP50-95)	0.295	0.168	0.243	0.268	0.275	0.279

Given the more significant number of boxes with infected leaves (7602) compared to grapes (2792), the model achieves higher mAP50 and mAP50-95 scores for unhealthy leaves across all image processing techniques. The difference is not major, indicating that the grape detection task is of lower complexity. This could be due to more distinctive and uniform features of grapes that the model can learn, even at lower resolutions.

The last two columns from Table 6 show that this dataset's performances can be increased using larger model architectures. For example, YOLO8-nano has only 3.2 M params and reaches for the task of unhealthy leave detection, using the *swds640* preprocessing, a MAP50 of 0.477 compared to the YOLO8-small (11.2 M params) with a MAP50 of 0.523, YOLO8-medium (25.9 M params) with a MAP50 of 0.533 and YOLO8-large (43.7 M params) with a MAP50 of 0.540.

Comparing experiments *split1* (Table 6 and Table 5-first experiment column) with *split2* (Table 8 and Table 5-second experiment column) one can infer that a non-uniform and non-continuous splitting of data in train and test dataset (i.e. taking also intermediate frames from videos to the testing dataset not just the last frames of each video) reflects in better model performances.

**Figure 8:**
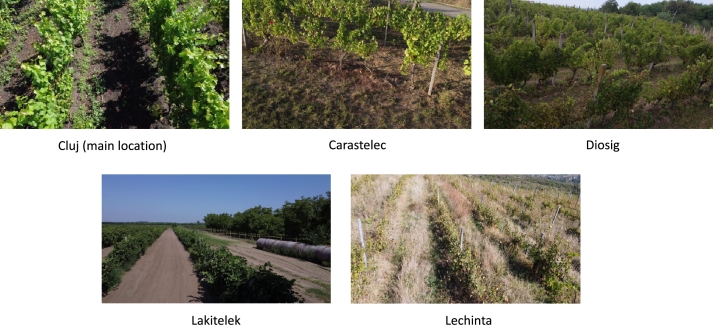
Samples of captures from different locations.

From Table 7, one may conclude that the results vary from one location to another, indicating the generalization ability is highly dependent on the characteristics of the training data. As a remark, for some of the locations where the performances are weaker, the drone flew a bit higher (Apoldu, Carastelec), the pictures were taken from different angles (Diosig), the camera was oriented too high (Ilok, Diosig, Lakitelek), or the landscapes vary much (Lechinta) from the acquisition from the main location. Samples from these locations are illustrated in Fig. 8. There are evident trade-offs between precision and recall in various locations, indicating that the model may be optimized towards one metric over the other. For instance, locations with high accuracy but low recall (e.g., Bontida, Lechinta, Simleu) suggest the model is conservative in its predictions, prioritizing correctness over completeness. Conversely, locations with higher recall but lower precision (e.g., Carastelec, Kecskemet) indicate the model detects more true positives but at the cost of more false positives.

**Table 7:** Model trained on all data from Cluj and evaluated on the other locations on the task of unhealthy leaves. The results are reported for all experiments image processing experiments: full size 4k, sliding windows and downscale, and downscale only.

	4k				swds640		ds640	
**Location**	**Prec.**	**Rec.**	**mAP50**	**mAP50-95**	**mAP50**	**mAP50-95**	**mAP50**	**mAP50-95**
Apoldu	0.442	0.219	0.246	0.119	0.228	0.160	0.0538	0.03
Beltiug	-	-	-	-	-	-	0	0
Bontida	0.714	0.429	0.409	0.271	0.471	0.311	0.0846	0.0665
Bordeaux	0.244	0.297	0.200	0.101	0.107	0.051	0.0687	0.0298
Carastelec	0.180	0.385	0.106	0.049	0.056	0.040	0.00852	0.00265
Diosig	0.280	0.272	0.179	0.087	0.104	0.053	0.0487	0.0217
Kecskemet	0.212	0.366	0.195	0.096	0.180	0.098	0.0288	0.01
Lakitelek	0.928	1.000	0.995	0.497	0.995	0.398	0.0553	0.0276
Lechinta	0.326	0.267	0.215	0.104	0.109	0.043	0.0642	0.0267
Simleu	0.939	0.286	0.427	0.224	0.572	0.226	0.0153	0.00617
Szekkutas	0.290	0.289	0.227	0.117	0.129	0.049	0.0882	0.0415
Szucsi	0.454	0.424	0.391	0.207	0.342	0.172	0.169	0.0787

Moreover, in this scenario, utilizing native 4K images for YOLOv8 (Table 9) or using sliding windows for the training experiments typically yielded superior outcomes in identifying unhealthy leaves and grapes when contrasted with the results from using downscaled versions.

Rows 3 and 4 of Table 6, Table 8, Table 9 present the performance of the detection method using the YOLOv8 models for grape identification. This allows us to compare and contrast the efficacy of different preprocessing techniques across varied tasks in agricultural image analysis. The experiment *split1* shows that the preprocessing methods, including direct downsampling (ds640) and the sliding window approach (swds), significantly impact model performance. Using the segmentation approach (Table 10), better results are obtained for both splittings and image sizes. The sliding window approach, which effectively increases the number of training and test images from 7432 to 29728, provides more detailed and varied data for the model to learn from, compared to direct downscaling.

**Table 8:** YOLOv8-split2 (detection): 4K - 3840x2160; ds640 - 4k downsampled to 640x360; swds640 - sliding window 4K to 1920x1080 then downsampled to 640x360.

	4k	ds640	swds640
train images	7979	7979	31916
test images	1995	1995	7980
grape (MAP50)	0.479	0.104	0.275
grape (MAP50-95)	0.199	0.031	0.113
unhealthy (MAP50)	0.584	0.383	0.517
unhealthy (MAP50-95)	0.306	0.18	0.264

**Table 9:** YOLOv8-split3 (detection): 4K - 3840x2160; ds640 - 4k downsampled to 640x360; swds640 - sliding window 4K to 1920x1080 then downsampled to 640x360.

	4k	ds640	swds640
train images	8955	8955	35820
test images	1019	1019	4076
grape (MAP50)	0.346	0.012	0.038
grape (MAP50-95)	0.141	0.005	0.017
unhealthy (MAP50)	0.318	0.07	0.279
unhealthy (MAP50-95)	0.157	0.031	0.140

**Table 10:** Results on the grape segmentation approach using the YOLOv8 nano network. There are also used various image sizes and sliding windows approach: ds640 - 4k downsampled to 640x360; swds640 - sliding window 4K to 1920x1080 then downsampled to 640x360.

	*split1*		*split2*	
	ds640	swds640	ds640	swds640
train images	7510	30031	7983	31923
test images	2470	9871	1997	7979
map50	0.084	0.169	0.111	0.326
map50-95	0.027	0.057	0.033	0.12

**Figure 9:**
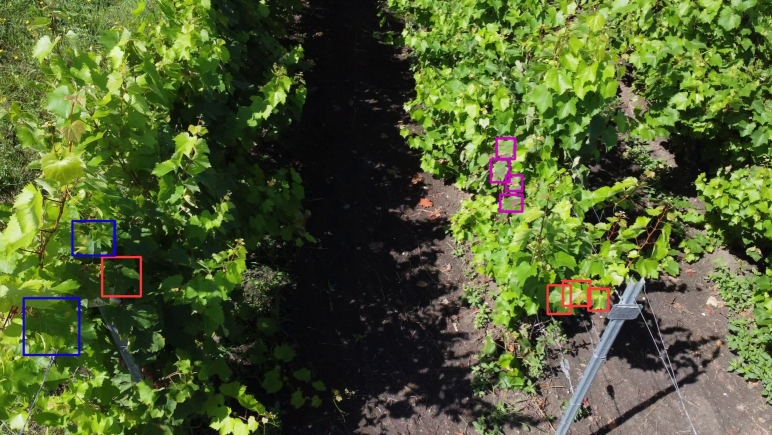
Example of the vine disease detection (YOLOv8, 4K). Red - correct, Blue - false negative, Purple - false positive.

An example showing also the limitations of the detection can be found in Fig. 9. As can be observed, the confidence of the detection decreases for the leaves further away from the camera, especially for the false negative alarms. These methods were trained on a server-grade computer, and the results are backed up using the Roboflow API, where the advantage is low-effort deployment on embedded devices.

### 3.2 Deployment results on embedded devices

Multiple detections were made on Android/IOS using the Roboflow API. For the Android side, another good result was obtained on the phone with Android 13 with Qualcomm SM8150 Snapdragon 855 4G, where the detection ran between 7-12 fps, which gave us a relatively good view of the possibility of implementing our model on a low-end device. On the IOS side, the best results were processed using an IOS device with an A16 Bionic chipset, which runs between 12-18 fps with the downsampled images. The full-size images could be evaluated at 10 fps using an Nvidia-enhanced AGX Jetson platform. Furthermore, testing the deployment on native and optimized models on Android and Jetson platforms gave us, on average, 10% better runtimes. With the onboard processing from the live camera feed, including the image transmission, preprocessing, and detection, achieving above 1 fps frame rates on mid-range mobile devices.

### 3.3 Discussion

Climate change will pose a significant challenge for grape growers in the coming decades. Europe, where some of the most famous wine regions are located, underwent significant temperature fluctuations in the 21st century. The global temperature data records since 1850 indicate that 2023 was the hottest calendar year, exceeding 2 ^∘^C in July and August, as the European Center for Medium-Range Weather Forecasts (ECMWF) reported. These agricultural regions are affected by climate change, mainly by rising ambient temperatures, heat waves, and weather variability, which entail decreases in general precipitation. Furthermore, they experience torrential rains, hail, and prolonged and severe droughts that jeopardize grape yield and quality [68]. Modern society shows growing concern and interest in environmental and human health. The chemical industry is a major and pervasive source of global environmental pollution. Pesticides protect crops and deter vector-borne diseases. However, they can damage agricultural and natural ecosystems [69], [70]. The sustainability of the winemaking industry can be improved by using AI-based technologies. With the help of these, pesticide usage, resource consumption, and waste generation can be reduced. AI algorithms can provide valuable data to help decrease pesticide pollution and water and nutrient inputs for grape cultivation, resulting in higher quality yields and lower environmental impact.

This study investigated five parasitic infections. Other studies identified *Flavescence dorée*, Esca and grapevine leafroll associated virus (GLRaV) in grapes [8], [25], [71], [72], [73], [74]. Hence, other diseases and stresses require further examination. Furthermore, symptomatic and asymptomatic hosts should be detected for damage, particularly when symptoms are indistinct. The disease history of the USAMV's vineyard was essential to avoid misclassifying asymptomatic plants as healthy plants. This article yielded useful results that suggest new opportunities to apply remote sensing technologies to precision viticulture. UAV features can be readily used to research and plan vineyard disease control strategies, including low-cost, timely delivery of high-resolution images and flexible flight planning. This section will address the following aspects of working on the vineyard analysis task: data collection, data diversity, and various aspects of automated detection using machine learning, such as input size, image processing, and augmentation.

Vineyard data can be collected from various sources, depending on the landscape accessibility, lighting, and weather conditions, as well as the detail level required by the task to be solved. Drones flying in vineyard analysis become more popular in the research community [29], [75], [76]. The experiment performed in this work is drone-based, with RGB images acquired at 1 to 2 meters above the canopy. This method of capturing vineyard data is in line with the approach taken by [51]. However, it diverges from the methodology of [29], [55], [75], [77], which utilized higher-altitude flights, near-infrared sensing or combined captures from satellite images. This difference in approach highlights the balance between the need for detailed visual information and the broader landscape insights provided by NIR imagery. Similarly, [51] emphasizes the importance of dataset diversity and the role of UAV-based RGB data in enhancing disease detection capabilities, a principle that aligns with the data collection strategy presented in this paper.

Downsizing is widely used to manage computational complexity and ensure that models can be effectively trained [54]. For YOLOv8n, training on native 4K images generally provided better results for detecting unhealthy leaves and grapes than downscaled versions. This suggests that higher-resolution images, despite their computational challenges, contain critical information for detecting small or subtle features. As demonstrated in this study, the significance of pre-processing techniques echoes the findings of [29], where image resolution was crucial to accurately identifying vineyard diseases. The work of [12], [13], [38] further supports the need for high-quality, diverse datasets to train robust models, underscoring the value of the sliding window approach in increasing the size and variety of the dataset.

Another important conclusion is that not only the number of images is important but also the number of detection samples. In the case of grape detection, there are only 2792 boxes, compared to 7602 for infected leaves (see Table 4), hence better results for the latter task.

The challenge of detecting specific diseases, such as downy mildew, as explored in [13], or for Botrytis cinerea as in [55] provides a valuable context for the focus of the study on infected leaf detection. The comparative advantage of the presented methodology may lie in the combination of high-resolution imagery and the comprehensive nature of the published dataset, which could offer improved detection rates compared to the scenarios presented in these referenced studies.

As suggested in Table 7, location-specific models or adaptations might be necessary to achieve uniformly high performance in different geographical areas. A mixed training set can be used as a future improvement but with a balanced number of captures from all locations. The generalization capability of models finds a parallel in the work presented by [46], [52], [77], where the adaptability of machine learning models to diverse environmental conditions was a key focus. The variance in model performance across different locations underscores the need for adaptable, location-specific models, a challenge that is also reflected in the broader literature. As in our experiment, [54] varieties the acquisitions across different phenological stages.

To sum up, the main challenges were to balance the image processing techniques and model architecture to deal with the small area of infected leaves and the diversity of the infection appearance, which may be easily confused with a young vine shoot or background. Another major issue is the method's generalisation and extrapolating it to different geographical areas. A high-accuracy model for downy mildew, powdery mildew, black rot, excoriose, and anthracnose symptoms recognition is an initial step for developing an automatic detection tool, but it is inadequate alone. Artificial intelligence tools should have transparent, understandable, and explainable results and processes in viticulture and other domains with social and environmental implications.

In the future, the integration of mixed training sets, as suggested in the conclusions, aligns with the recommendations of [75], [52], and [51] to improve model robustness and generalization capabilities. This approach and the potential exploration of multi-spectral imaging techniques and advanced neural network architectures present a promising avenue for future research. Another point of interest is to apply a segmentation algorithm or model and work only with the vineyard pixels so that the background will not negatively influence the prediction.

The deployment of unmanned aerial vehicle (UAV) technology for the proactive detection and ongoing surveillance of pathogenic threats enables the gathering of essential data, such as acquisitions of photos/videos associated with GPS coordinates. This information is crucial for enhancing viticultural practices, improving efficiency over time, and increasing agricultural output. The technical proficiency and training required to operate UAVs present a challenge for some farmers in effectively employing drones for plant disease management. For example, high initial costs, specialized human resources, strong wind or other adverse weather conditions, lack of electricity, etc. This challenge may necessitate further investment in training and educational programs to ensure farmers' safe and proficient use of UAVs. Additionally, our analysis indicates that research on UAV-based plant disease detection and monitoring methods is not uniformly distributed worldwide. This underscores the necessity for additional evaluations of UAV systems in various agricultural contexts to determine their efficacy and adaptability to different crops, climatic conditions, and terrains. The uneven global distribution of research on UAV-based plant disease monitoring also suggests that such research within a country does not necessarily indicate the nation's scientific and technical capabilities or economic status.

Economic and accessibility concerns related to advanced drone technology and complex software remain significant barriers to adopting autonomous UAVs. Nevertheless, there is a growing trend in the industry towards reducing the costs associated with drone technology and making it more user-friendly. Efforts are underway to develop drones with intuitive controls and automated features that simplify operations. Simultaneously, software enhancements aim to provide more user-friendly interfaces and efficient data management tools. These developments are crucial in making autonomous UAVs more accessible and practical for a broad range of uses, enabling their benefits to be leveraged across various sectors of society.

## 4 Conclusion

This work proposes a novel method of monitoring vine disease based on proximity image processing from low-altitude UAVs. An entire season-based data acquisition campaign was provided, which captured different periods of the year when various plant structures and diseases affect the plants. The vine disease dataset can be used for generic disease detection by providing a large and generic dataset that covers more than 12 regions in five countries and more than 100,000 images over a whole season. The over 100,000 labelled images were validated by human experts. Two well-suited methods were evaluated for different set-ups, including large and small optimal detection and data transmission resolutions. Furthermore, the proposed models were deployed on several embedded devices, highlighting the applicability of the proposed pipeline.

Due to the focus of this dataset on fungi and bacteria-caused diseases, it can be refined in the future by marking different classes for certain diseases, such as pest—and insect-based ones, phylloxera, or mites, as observed in a few images. In addition, at the same time as our flight campaigns, several photographs were taken using handheld smartphones and a robot-mounted DSLR camera, which are planned to be annotated and published.

The incorporation of drone technology in evaluating grape vine pathologies affords a proficient means of surveillance and identification that is pivotal for advancing intelligent agriculture. Drones enhance reachability, extend the examination scope, and expedite data aggregation, facilitating the prompt discernment of diseases. The assimilation of such data, when analyzed through computational analytics and machine learning paradigms, allows the delineation of disease patterns and the evaluation of their intensity. Integrating drone systems within plant disease assessment frameworks enables continuous monitoring, intelligent detection, and precise management. Drones are instrumental in promoting sustainable agronomic practices, decreasing crop yield, reducing reliance on chemical interventions, and bolstering precision farming methodologies. The initial costs of integrating the UAV-based monitoring can be assimilated during the low-cost usage of these systems, especially with the lack of human workforce in viticulture.

In the future, the integration of mixed training sets, as suggested in the conclusions, aligns with the recommendations of [75], [52], and [51] to improve model robustness and generalization capabilities. This approach and the potential exploration of multi-spectral imaging techniques and advanced neural network architectures present a promising avenue for future research. Another point of interest is to apply a segmentation algorithm or model and work only with the vineyard pixels so that the background will not negatively influence the prediction.

The deployment of unmanned aerial vehicle (UAV) technology for the proactive detection and ongoing surveillance of pathogenic threats enables the gathering of essential data, such as acquisitions of photos/videos associated with GPS coordinates. This information is crucial for enhancing viticultural practices, improving efficiency over time, and increasing agricultural output.

The technical proficiency and training required to operate UAVs present a challenge for some farmers in effectively employing drones for plant disease management. For example, high initial costs, specialized human resources, strong wind or other adverse weather conditions, lack of electricity, etc. This challenge may necessitate further investment in training and educational programs to ensure farmers' safe and proficient use of UAVs. Additionally, our analysis indicates that research on UAV-based plant disease detection and monitoring methods is not uniformly distributed worldwide. This underscores the necessity for additional evaluations of UAV systems in various agricultural contexts to determine their efficacy and adaptability to different crops, climatic conditions, and terrains. The uneven global distribution of research on UAV-based plant disease monitoring also suggests that such research within a country does not necessarily indicate the nation's scientific and technical capabilities or economic status.

Economic and accessibility concerns related to advanced drone technology and complex software remain significant barriers to adopting autonomous UAVs. Nevertheless, there is a growing trend in the industry towards reducing the costs associated with drone technology and making it more user-friendly. Efforts are underway to develop drones with intuitive controls and automated features that simplify operations. Simultaneously, software enhancements aim to provide more user-friendly interfaces and efficient data management tools. These developments are crucial in making autonomous UAVs more accessible and practical for a broad range of uses, enabling their benefits to be leveraged across various sectors of society.

## Funding

The authors are thankful for the DGX grade server offered by NVidia to support this work. This work was also financially supported by the Romanian 10.13039/501100005802National Authority for Scientific Research, project nr. PN-III-P2-2.1-PED-2021-3120.

## CRediT authorship contribution statement

**Delia Elena Székely:** Validation, Methodology, Formal analysis, Data curation. **Darius Dobra:** Data curation. **Alexandra Elena Dobre:** Visualization, Data curation. **Victor Domşa:** Visualization, Software, Data curation. **Bogdan Gabriel Drăghici:** Software, Investigation, Data curation. **Tudor-Alexandru Ileni:** Writing – original draft, Validation, Data curation. **Robert Konievic:** Investigation, Data curation. **Szilárd Molnár:** Writing – original draft, Visualization, Validation, Software, Data curation. **Paul Sucala:** Validation, Data curation, Conceptualization. **Elena Zah:** Visualization, Data curation. **Adrian Sergiu Darabant:** Writing – review & editing, Supervision, Project administration, Conceptualization. **Attila Sándor:** Project administration, Investigation, Conceptualization. **Levente Tamás:** Writing – review & editing, Supervision, Methodology, Funding acquisition.

## Declaration of Competing Interest

The authors declare the following financial interests/personal relationships which may be considered as potential competing interests: Levente Tamas reports was provided by Technical University of Cluj-Napoca. Levente Tamas reports a relationship with Technical University of Cluj-Napoca that includes: employment. If there are other authors, they declare that they have no known competing financial interests or personal relationships that could have appeared to influence the work reported in this paper.

## Data Availability

The datasets generated during and/or analysed during the current study are available from the corresponding author on reasonable request.

## Footnotes

3 https://github.com/tamaslevente/vineye.

4 https://www.cvat.ai/.

5 https://github.com/openvinotoolkit/datumaro.

6 https://roboflow.com/.

7 https://github.com/tamaslevente/vineye.
